# Ketosis Home Management in Pediatric Type 1 Diabetes in Germany: Mismatch Between Subjective Self-Ratings and Objectively Assessed Competence in Preventing Diabetic Ketoacidosis

**DOI:** 10.3390/children13050592

**Published:** 2026-04-24

**Authors:** Simone Eisenhofer, Martina Patrizia Neininger, Astrid Bertsche, Wieland Kiess, Thilo Bertsche, Thomas Michael Kapellen

**Affiliations:** 1 Drug Safety Center, Leipzig University Hospital, Leipzig University, Brüderstrasse 32, 04103 Leipzig, Germany; simone.eisenhofer@uni-leipzig.de (S.E.); martina.neininger@uni-leipzig.de (M.P.N.); 2 Clinical Pharmacy, Institute of Pharmacy, Medical Faculty, Leipzig University, Brüderstrasse 32, 04103 Leipzig, Germany; 3 Department of Neuropediatrics and Metabolic Diseases, University Medicine Greifswald, Ferdinand-Sauerbruch-Strasse 1, 17475 Greifswald, Germany; astrid.bertsche@uni-greifswald.de; 4 Center for Pediatric Research, University Hospital for Children and Adolescents, Liebigstrasse 20a, 04103 Leipzig, Germany; wieland.kiess@medizin.uni-leipzig.de (W.K.); thomasmichael.kapellen@medizin.uni-leipzig.de (T.M.K.)

**Keywords:** diabetes mellitus, type 1 diabetes (T1D), diabetic ketoacidosis (DKA), diabetic ketosis, insulin administration, ketone testing, sick-day management, urine dipsticks

## Abstract

**Highlights:**

**What are the main findings?**

- Adolescents and caregivers of younger children living with T1D subjectively self-rated their competence in ketosis home management aimed at preventing diabetic ketoacidosis (DKA) as good to very good.
- The objective assessment revealed challenges in the home management of ketosis, which may compromise glycemic control and thereby hinder the prevention of DKA.

**What are the implications of the main findings?**

- Our study revealed a mismatch between challenges demonstrated in the objective assessment and the high subjective self-ratings of competence in ketosis home management.
- To overcome this potential barrier to ideal glycemic control, recurrent individualized hands-on training, including clinical case scenarios and simulation-based demonstrations, as well as comprehensive ketosis/DKA awareness campaigns are needed.

**Abstract:**

Background: Effective sick-day management, including ketosis home management aimed at preventing diabetic ketoacidosis (DKA), is essential for families living with a child/adolescent with type 1 diabetes (T1D). Methods: Adolescents living with T1D and caregivers of younger children living with T1D were invited to participate in an interview consisting of five parts: (I) demographic data, (II) subjective self-ratings on competence in ketosis home management, (III) objective assessment of competence in ketosis home management using a standardized clinical case scenario consisting of 10 management steps, in which participants were asked to describe the actions they would take to prevent DKA, and (IV) practical demonstrations to objectively assess skills in (IVa) urine dipstick self-testing and (IVb) insulin administration, (V) household availability of (Va) urine dipsticks and (Vb) insulin cartridges. Results: (I) We enrolled 61 adolescents and 79 caregivers. (II) Competence in ketosis home management was subjectively self-rated as good to very good. (III) Adolescents reported 4 (median; Q25/Q75 3/5) and caregivers 5 (4/5) of 10 management steps. Never self-testing ketone levels was reported by 33% of adolescents and 11% of caregivers. (IVa) At least one handling error occurred in 100% of adolescents’ and in 98% of caregivers’ practical demonstrations of urine dipstick self-testing and in (IVb) 98% of adolescents’ and 98% of caregivers’ insulin administrations. (Va) Altogether urine dipsticks were available in 43% of households, whereas (Vb) insulin cartridges were available in 78% of households. Conclusions: Our results demonstrate a mismatch between challenges in ketosis home management and high subjective self-ratings.

## 1. Introduction

Type 1 diabetes (T1D), the most common metabolic disorder in pediatrics, is associated with potentially life-threatening glycemic emergencies [1,2,3]. The risk of metabolic decompensation increases during acute illness, highlighting the importance of comprehensive sick-day management training for individuals living with T1D [2,4,5]. A fundamental aspect of sick-day management is preventing diabetic ketoacidosis (DKA), which is characterized by hyperglycemia, metabolic acidosis, ketonemia and ketonuria [2,4,6]. DKA is a serious acute complication and the leading cause of diabetes-related mortality and morbidity in pediatrics [3,4,6,7]. The risk of developing DKA is highly individual and ranges from 1 to 10% per patient-year [4].

DKA-related hospitalization is associated with adverse long-term health outcomes and should therefore be prevented at an early stage [6,8,9,10,11]. Effective prevention strategies include the early recognition of diabetic ketosis and therefore impending DKA, ketone self-testing, the calculation and administration of supplemental insulin, the monitoring of blood glucose and ketone levels, and knowledge on when and how to seek medical assistance [2,6,12]. In this publication, these patient-led measures aimed at preventing DKA are referred to as ketosis home management.

The management of pediatric T1D always includes caregivers and other family members [4]. However, families living with T1D often struggle to apply sick-day management strategies, particularly those related to ketosis and DKA [13,14,15,16]. Most previous studies have relied on self-administered questionnaires and quizzes. So far, little research has employed clinical case scenarios or practical demonstrations to assess ketosis home management and DKA prevention strategies.

In this exploratory study, we objectively assessed challenges of adolescents living with T1D and caregivers of younger children living with T1D in ketosis home management, ketone self-testing with urine dipsticks, and insulin administration using a standardized clinical case scenario and practical demonstrations. We subsequently compared these objectively assessed findings to participants’ corresponding subjective self-ratings.

## 2. Materials and Methods

### 2.1. Participants and Setting

**Figure 1:**
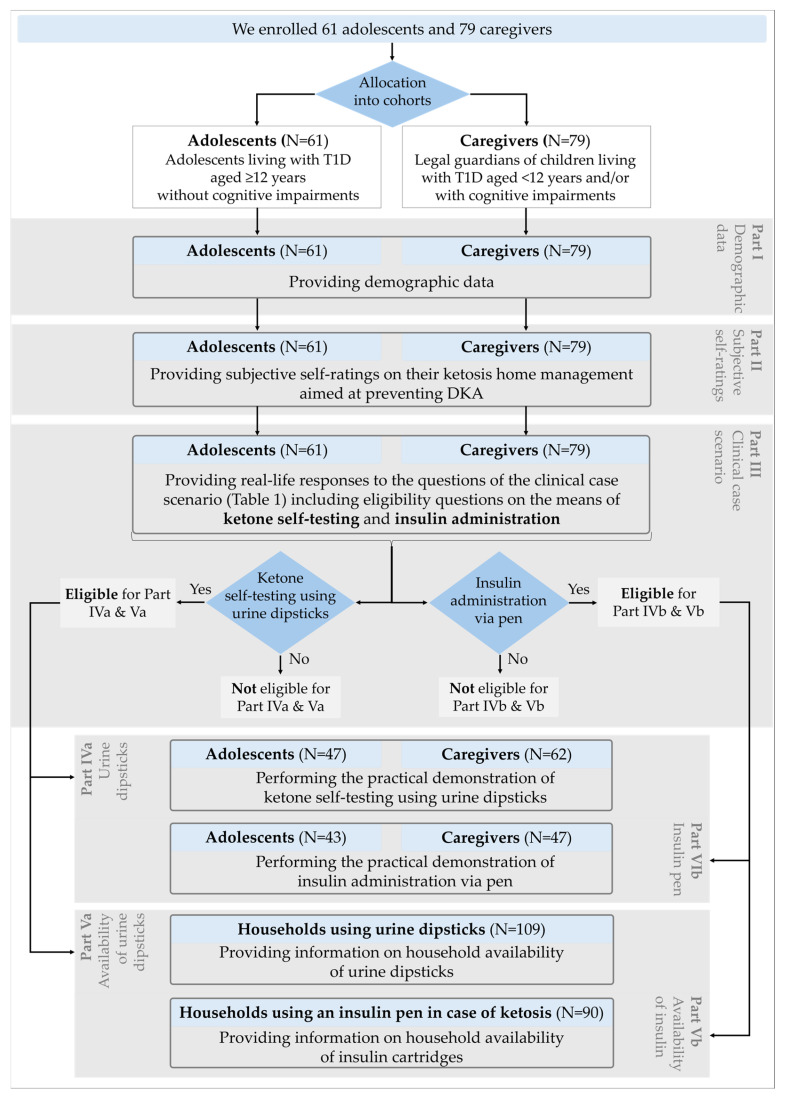
Study flow chart including parts of the standardized semi-structured interview. DKA: Diabetic ketoacidosis. T1D: Type 1 diabetes.

Adolescents living with T1D and caregivers of younger children living with T1D attending outpatient appointments at the pediatric diabetes department were consecutively invited to participate in this exploratory study. Although adolescents aged 12 years and older without cognitive impairment are not expected to manage diabetic ketosis on their own, they should be educated about ketosis home management and the prevention of DKA [17]. Accordingly, these adolescents were enrolled in the “adolescents” cohort. Caregivers of younger children living with T1D or of adolescents with cognitive impairment were enrolled in the “caregivers” cohort (Figure 1). Consequently, for each data collection, only the adolescent or the caregiver was enrolled, never both.

The inclusion criteria were as follows:

- Adolescents were:
  ○ Diagnosed with T1D in accordance with the national guideline [4];
  ○ Aged ≥12–<18 years;
  ○ Generally healthy without cognitive impairment;
  ○ Proficient in the German language.
- Caregivers were:
  ○ Legal guardians of a child living with T1D younger than 12 years old or of an adolescent with cognitive impairment;
  ○ Proficient in the German language;
  ○ The caregiver most involved in diabetes care.

Participants with multiple routine appointments during the study period were enrolled only once. We obtained written informed consent from all enrolled caregivers, from the legal guardians of enrolled adolescents and from adolescents aged 14 years or older, in accordance with legal requirements.

Interview Section 3.1 and Section 3.2 (I: demographic data, II: subjective self-ratings) were performed after enrollment at the pediatric diabetes department. To observe the real-life therapy environment, we aimed at conducting Section 3.3, Section 3.4 and Section 3.5 (III: standardized clinical case scenario, IV: practical demonstrations, V: household availability of urine dipsticks and insulin cartridges) at participants’ homes (Figure 1). If home visits were refused, Section 3.3 and Section 3.4 were conducted at the pediatric diabetes department and Section 3.5 was conducted via telephone, enabling participants to refer to storage and expiration details.

### 2.2. Standardized Semi-Structured Interview

An expert panel of pharmacists, pediatricians, and pediatric diabetologists developed a standardized semi-structured interview questionnaire based on the following sources:

- Ketosis/DKA prevention training program and ketosis management plan of the pediatric diabetes department where the study was conducted.
- National and international DKA guidelines [2,3,4,6,7,12,18].
- The package insert of the commonly used urine dipsticks (Keto-Diastix Reagent Strips, Ascensia Diabetes Care Holdings AG, Leverkusen Germany) [19].
- Summaries of product characteristics of commonly used insulins (NovoRapid, Novo Nordisk Pharma GmbH, Mainz, Germany; Liprolog, Eli Lilly Nederland B.V., Utrecht, The Netherlands; Insulin lispro Sanofi, Sanofi-aventis Groupe, Paris, France; HUMALOG, Eli Lilly Nederland B.V., Utrecht, The Netherlands) [20,21,22,23].
- The “Guideline on Injections in Diabetes Mellitus” issued by the Association of Diabetes Counseling and Training Professions in Germany regarding injection techniques for individuals living with diabetes mellitus [24].

As the local training protocol did not differentiate between diabetic ketosis and DKA, we referred to both conditions as DKA when addressing participants.

We pretested the standardized semi-structured interview with 5 pharmacists not involved in the study and piloted it with 11 participants. Following minor adjustments in the pilot phase, the pilot data were included in the study. A trained pharmacist conducted all interviews to ensure consistency and minimize interviewer bias.

The semi-structured interview consisted of 5 parts (Figure 1):

Part I: Demographic data

We obtained the following demographic data: age, gender, education level, and profession. Missing information was obtained from medical records. Participants were also asked when they last attended a training session on ketosis and DKA prevention.

Part II: Subjective self-ratings on competence in ketosis home management aimed at preventing DKA

Participants were asked to provide subjective self-ratings on the topics “Knowledge of the concept of DKA”, “ability to identify symptoms of DKA”, and “skills in managing DKA” using a scale ranging from very poor (1), inadequate (2), acceptable (3), fair (4), good (5), to very good (6). A score of “non-existent (0)” was assigned to participants who reported that they did not know what DKA was. Participants were asked to indicate their desire for ketosis/DKA prevention training using the following scale: no desire (0), little desire (1), moderate desire (2), great desire (3).

Part III: Standardized clinical case scenario to objectively assess competence in ketosis home management aimed at preventing DKA

Competence in ketosis home management aimed at preventing DKA was objectively assessed using a standardized clinical case scenario addressing 10 management steps in 3 domains: initiation, implementation and termination of ketosis home management (Table 1). Participants were asked to describe their real-life responses to the questions of the standardized clinical case scenario, which were then compared to recommendations in national and international guidelines [2,3,4,6,7,12,18].

Guidelines differ in their first-line recommendations for calculating increased insulin doses to prevent DKA [2,4]. In the study setting, participants were trained according to the German guideline, which recommends doubling the usual correction bolus. This approach is considered feasible by international standards [2,4]. Management step 2 (calculate the increased insulin dose) was therefore used to assess adherence to this approach. Management step 9 (mandatory: seek immediate medical assistance if ketone and/or blood glucose levels remain elevated) served as a red-flag criterion to assess whether participants recognize the need for immediate medical assistance when ketosis home management proves insufficient. Regardless of management step 9, any reports of seeking assistance during the standardized clinical case scenario were documented.

If participants referred to the ketosis management plan they had received during training at the pediatric diabetes department, the plan was provided for them as guidance during the standardized clinical case scenario. Participants were also asked which circumstances would prompt initial ketone self-testing. Any additional ketosis- and/or DKA-related measures reported spontaneously by the participants were documented.

Part IV: Practical demonstrations to objectively assess skills in (IVa) urine dipsticks self-testing and (IVb) insulin administration

Participants who reported using urine dipsticks for ketone self-testing during the standardized clinical case scenario were eligible for Part IVa (Figure 1). They were asked to demonstrate self-testing with original urine dipsticks, including the package insert and a test solution imitating urine. Participants who reported using an insulin pen in case of diabetic ketosis during the standardized clinical case scenario were eligible for Part IVb (Figure 1). They were asked to demonstrate insulin administration using a dummy of the pen (no needle, placebo solution) that they were accustomed to using.

A trained pharmacist assessed both demonstrations and evaluated any handling errors based on the instructions of the respective package inserts and/or summary of product characteristics and insulin administration guidelines [19,20,21,22,23].

Part V: Evaluation of household availability of (Va) urine dipsticks and (Vb) insulin cartridges

Participants who reported using urine dipsticks and/or insulin pens during the standardized clinical case scenario were eligible for Part Va and/or Part Vb (Figure 1).

We evaluated the availability of usable urine dipsticks and/or insulin cartridges in participants’ households. If the data were collected during a home visit, the trained pharmacist verified the storage conditions and documented the expiration date. If the data were collected via telephone, participants described the storage conditions and read the expiration date to the trained pharmacist. Urine dipsticks were considered usable if they were within their expiration date, had been opened for no more than 6 months, and were available to the participant [19]. Similarly, insulin cartridges were considered usable if they were within their expiration date, stored correctly (2–8 °C), and available to the participant [20,21,22,23].

The availability of urine dipsticks and insulin cartridges was evaluated at the household level, as it could not be attributed exclusively to either adolescents or caregivers.

### 2.3. Statistics

Descriptive data are reported as numbers with corresponding percentages. For the presentation of continuous data, the medians including first and third quartile (Q25/Q75) and minimum/maximum are shown as appropriate. Excel (version 2411, Microsoft 365, Redmond, WA, USA) and Kyplot (version 6.0, KyensLab Inc., Tokyo, Japan) were used for statistical evaluation. We used Mann–Whitney U tests for group comparisons and considered *p* ≤ 0.05 to indicate significance.

### 2.4. Ethics Approval and Consent to Participate

This study was performed in line with the principles of the Declaration of Helsinki. Approval was granted by the Ethics Committee of the Medical Faculty at Leipzig University, Germany (Ethics approval number: 002/19-ek, approved 22 January 2019). We obtained written informed consent from every enrolled caregiver, from every legal guardian of enrolled adolescents, and from adolescents aged 14 years or older, in accordance with legal requirements. Trial registration number: DRKS00022341 (5 August 2020).

## 3. Results

### 3.1. Demographic Data

Demographic data of the 61 enrolled adolescents and 79 caregivers are presented in Table 2.

### 3.2. Subjective Self-Ratings on Competence in Ketosis Home Management Aimed at Preventing DKA

Participants’ self-ratings on ketosis home management competence ranged from good (5) to very good (6; Table 3). A score of “non-existent (0)” was assigned to 5/61 (8%) adolescents who indicated that they did not know what DKA was. Both adolescents (median 1; Q25/Q75 1/2; min/max 0/3) and caregivers (1; 0/2; 0/3) expressed low desire for ketosis/DKA prevention training.

### 3.3. Standardized Clinical Case Scenario to Objectively Assess Competence in Ketosis Home Management Aimed at Preventing DKA

**Figure 2:**
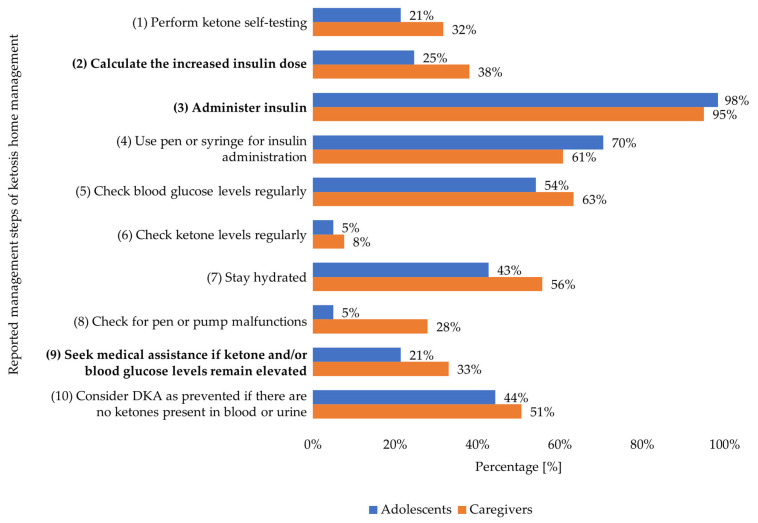
Reported management steps. DKA: Diabetic ketoacidosis. Highly critical management steps are highlighted in bold lettering. Management step 9: Red-flag criterion for the need for immediate medical assistance.

Of the 10 management steps used to objectively assess ketosis home management, adolescents reported 4 steps (median; Q25/Q75 3/5; min/max 1/8) and caregivers reported 5 steps (4/5; 1/9; Figure 2).

Regarding management step 1 (perform ketone self-testing), 10/61 (16%) adolescents and 17/79 (22%) caregivers reported to self-test in blood plasma, while 47/61 (77%) adolescents and 62/79 (78%) caregivers would use urine dipsticks, and 4/61 (7%) adolescents did not know how to test for ketones. Among participants using an insulin pump in regular diabetes therapy, 7/25 (28%) adolescents and 14/46 (30%) caregivers reported to switch to pen or syringe for insulin administration in case of diabetic ketosis (step 4). Management step 8 (check for pen or pump malfunctions) was exclusively reported by participants using an insulin pump in regular diabetes therapy [adolescents: 3/25 (12%); caregivers: 22/46 (48%)]. Regarding management step 9 (mandatory: seek immediate medical assistance if ketone and/or blood glucose levels remain elevated), 4/61 (7%) adolescents and 22/79 (28%) caregivers reported to contact the pediatric diabetes department, while 9/61 (15%) adolescents and 4/79 (5%) caregivers reported to initiate hospital admission.

The ketosis management plan was used by 11/61 (18%) adolescents and 19/79 (24%) caregivers. Participants who used the ketosis management plan (*n* = 30) reported significantly more management steps (median 5; Q25/Q75 4/6; min/max 2/9) than those who did not (*n* = 110; 4; 3/5; 1/8; *p* ≤0.001).

**Figure 3:**
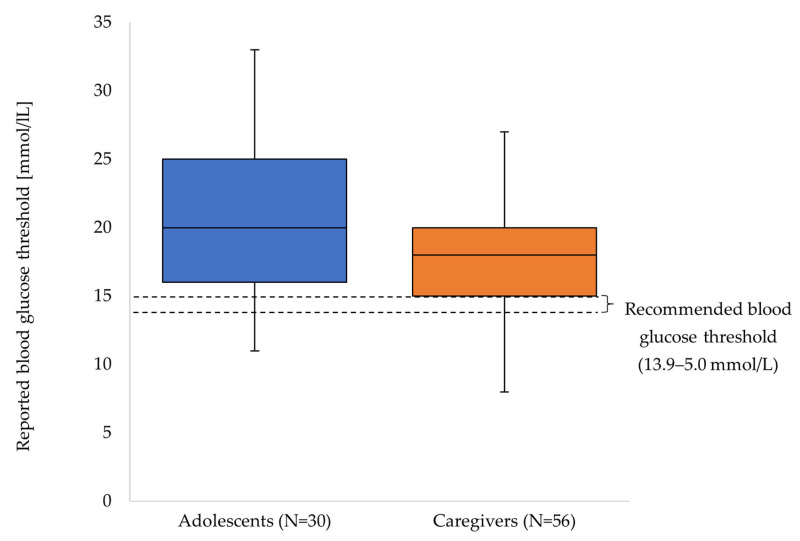
Reported blood glucose thresholds to prompt initial ketone self-testing.

When asked about circumstances that would prompt initial ketone self-testing in the presence of nausea and abdominal pain, 31/61 (51%) adolescents and 57/79 (72%) caregivers reported a specific blood glucose threshold (Figure 3). The recommended blood glucose threshold of 13.9–15.0 mmol/L was reported by 2/61 (3%) adolescents and 15/79 (19%) caregivers. Testing of ketone levels regardless of blood glucose level, but based on general physical condition, was reported by 4/61 (7%) adolescents and 8/79 (10%) caregivers. Never self-testing ketone levels was reported by 20/61 (33%) adolescents and 9/79 (11%) caregivers [missing information: 6/61 (10%) adolescents, 5/79 (6%) caregivers].

**Figure 4:**
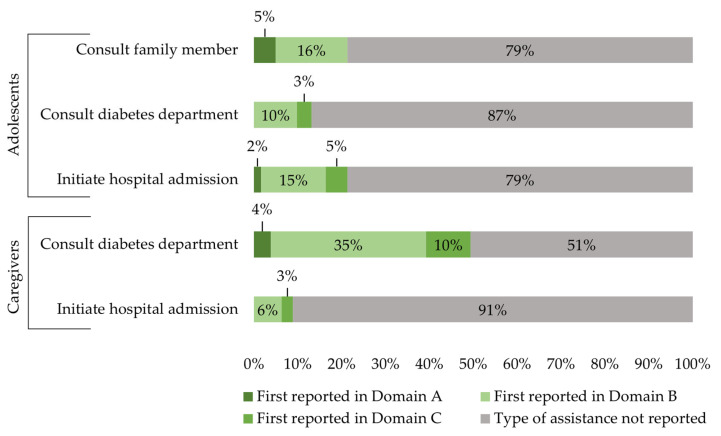
Type of medical or family-related assistance and the domain of the standardized clinical case scenario in which it was first reported. Medical assistance is considered optional in Domain A and B but mandatory in Domain C. Domain A: Initiation of ketosis home management. Domain B: Implementation of ketosis home management. Domain C: Termination of ketosis home management.

Throughout the standardized clinical case scenario, 41/61 (67%) adolescents and 36/79 (46%) caregivers did not report seeking medical assistance (Figure 4).

### 3.4. Practical Demonstrations to Objectively Assess Skills in (IVa) Urine Dipstick Self-Testing and (IVb) Insulin Administration

A total of 109 participants (47 adolescents, 62 caregivers) were enrolled in Part IVa, as they reported using urine test dipsticks during the standardized clinical case scenario. At least one handling error occurred in 107/109 (98%) of the practical demonstrations of urine dipstick self-testing, [adolescents: 47/47 (100%); caregivers: 60/62 (97%); Table 4]. Among participants who used the original package insert (*n* = 9) significantly fewer handling errors occurred (median 2; Q25/Q75 1/3; min/max 0/3) than among those who did not (*n* = 100; 3; 2/4; 0/5; *p* = 0.031).

A total of 90 participants (43 adolescents, 47 caregivers) were enrolled in Part IVb, as they reported using an insulin pen during the standardized clinical case scenario. The demonstration was refused by two adolescents and three caregivers. At least one handling error occurred in 83/85 (98%) demonstrations [adolescents: 40/41 (98%); caregivers 43/44 (98%); Table 5].

We found no significant difference (*p* = 0.499) in the median number of handling errors between participants who used an insulin pen for regular diabetes therapy (*n* = 65; 2; 1/4; 0/6) and those who used an insulin pump for regular diabetes therapy but would switch to an insulin pen in case of diabetic ketosis (*n* = 20; 2, 2/2, 0/5).

### 3.5. Evaluation of Household Availability of (Va) Urine Dipsticks and (Vb) Insulin Cartridges

A total of 109 households were enrolled in Part Va, as participants reported using urine test dipsticks during the standardized clinical case scenario. In 47/109 (43%) households, usable urine dipsticks were available. In 13/109 (12%) households, dipsticks were expired, in 24/109 (22%) household dipsticks had been opened for more than six months, and in 15/109 (14%) households no dipsticks were available. The data are missing for 10/109 (9%) households.

A total of 90 households were enrolled in Part Vb, as participants reported using an insulin pen during the standardized clinical case scenario. In 70/90 (78%) households, usable insulin cartridges were available. In 1/90 (1%) household cartridges were stored at an inappropriate temperature, in 2/90 (2%) households no cartridges were present, and for 17/90 (19%) households information was not provided.

## 4. Discussion

### 4.1. General Considerations

This exploratory study’s objective assessment using a standardized clinical case scenario (Section 3.3) revealed challenges in ketosis home management aimed at preventing DKA. Only half (caregivers) or fewer than half (adolescents) of the management steps addressed in the interview were reported by the respective participants. The relevance of ketone self-testing was frequently overlooked, and participants reported higher-than-recommended blood glucose thresholds to induce ketone self-testing. Practical demonstrations of urine dipstick self-testing (Section 3.4) and insulin pen administration (Section 3.4) were shown to be error-prone. In nearly all practical demonstrations at least one handling error occurred. These handling errors included those potentially leading to false-negative ketone tests and inadequate insulin absorption. Although participants were well-equipped with insulin cartridges, fewer than half had access to usable urine dipsticks (Section 3.5). The objectively assessed challenges in ketosis home management and practical demonstrations were contradicted by the participants’ high subjective self-ratings and low desire for ketosis/DKA prevention training (Section 3.2).

### 4.2. Deficiencies Towards Ketosis Home Management Aimed at Preventing DKA

By median, participants reported half (caregivers) or fewer than half (adolescents) of the management steps addressed in the standardized clinical case scenario. Two of the least reported steps were related to ketone self-testing: Fewer than 10% of both cohorts would perform ketone monitoring regularly. Furthermore, one third of adolescents and about 10% of caregivers reported to never test ketone levels. Reluctance towards ketone self-testing is known from the literature [25], but previous studies reported higher levels of awareness towards the importance of self-testing ketones, varying from 20 to 76% [26,27,28,29].

The median reported blood glucose threshold prompting ketone self-testing was 20.0 mmol/L (adolescents) and 18.0 mmol/L (caregivers), respectively. Guidelines recommend a threshold of 13.9–15.0 mmol/L, which was reported by fewer than 6% of adolescents and 10% of caregivers. According to Mackay et al., the most frequently reported blood glucose thresholds prompting ketone self-testing ranged from 14 to 16 mmol/L, which more closely approximates the guideline recommendations [27].

While almost all participants of this study knew that insulin administration was necessary, only about one quarter of participants calculated an adequately increased dose. Furthermore, essential measures such as ensuring hydration and regular blood glucose assessment were reported by only about half of our participants. Alshareef et al. investigated similar measures but found higher awareness levels towards insulin dose calculation, hydration and blood glucose monitoring [28].

This study’s objective assessment of ketosis home management aimed at preventing DKA by means of a standardized clinical case scenario revealed considerable challenges. Previous studies also showed deficits in preventing DKA, but mostly with less frequent difficulties compared to this study [13,14,16,28,30,31,32]. This discrepancy may be attributable to the reliance on self-reported questionnaires in the referenced studies rather than pharmacist-assessed clinical case scenarios. Therefore, questionnaire-based self-assessments may underestimate challenges in preventing DKA.

### 4.3. Deficiencies Towards Ketone Self-Testing

Approximately 80% of participants in our study reported relying on urine dipsticks. This may be attributed to urine dipsticks frequently being perceived as affordable and convenient to use, although handling errors occur even among health professionals [2,33].

Consistent with these findings, the practical demonstrations conducted in our study revealed that the use of urine dipsticks is highly error-prone. The literature on handling errors in the use of urine dipsticks is scarce. Consequently, their clinical relevance remains underexplored. Beyond potential technical mishandling, we identified handling errors that may lead to false-negative results: more than 60% of participants of both cohorts interpreted the test result after an inappropriate time. In addition, 57% of adolescents and 44% of caregivers did not reseal the dipstick container immediately, potentially compromising the functionality and leading to unreliable results in future ketone self-testing. This risk is aggravated by the finding that expired or excessively long-opened test strips were present in more than one third of participants’ households, representing a further risk factor for false-negative or delayed ketone detection. Taken together, our findings provide further support for current guideline recommendations favoring blood ketone measurement or the use of continuous ketone monitoring systems [2,3,4,34,35,36].

### 4.4. Deficiencies Towards Insulin Administration

To identify the cause of diabetic ketosis and prevent further events, it is essential for adolescents and caregivers to check for device malfunctions [2,26]. Regarding regular pump users, only 12% of adolescents and almost half of caregivers reported examining the pump for improper function. On top of that, none of the regular pen users reported that they inspect if a pen malfunction caused diabetic ketosis. Because of potential malfunctions, pump use is not recommended for DKA prevention [2,6,26]. However, about 70% of this study’s participants using a pump in regular diabetes therapy would continue to use it in ketosis home management. This proportion is even surpassed by the 90% reliance on pumps reported in the literature [26].

This potential overconfidence in device functionality was also shown in the practical demonstrations of insulin administration: more than 90% of participants performed either no pen safety test or an incorrect one prior to insulin administration. In addition, we identified handling errors that would result in failure of insulin delivery: approximately one third of participants in both cohorts did not remove the needle cap prior to demonstrating the injection, and 27% of adolescents and 14% of caregivers failed to attach the (dummy) needle to the pen.

This risk of inadequate insulin administration and consequently potential hospitalization is further aggravated by the finding that approximately 50% of adolescents and more than 30% of caregivers withdrew the needle too quickly from the subcutaneous tissue. Although in an encouragingly high number of households usable insulin cartridges were available, 39% (adolescents) and 27% (caregivers) of those surveyed did not remove the needle from the pen after injection, thereby increasing the risk of microbiological contamination during storage. Furthermore, this may increase the likelihood of needle reuse in future insulin administration, thereby raising the risk of tissue damage and lipodystrophy [37]. These findings are consistent with questionnaire-based findings reporting similar, although generally less frequently observed, deficits in insulin administration [38].

Unexpectedly, no difference in the overall frequency of handling errors was observed between participants who reported using an insulin pen only in the rare event of pending DKA and those who used it as part of their daily diabetes therapy. This study focused on the prevention of DKA rather than daily diabetes therapy. However, the findings may indicate challenges in insulin administration among regular pen users, consistent with previous studies [38,39,40,41]. The high number of handling errors observed in our study further underscores the classification of insulin as a high-risk medication [42]. To reduce handling errors and improve glycemic control, pharmacist-led interventions focusing on correct pen use should be offered to families of children or adolescents living with T1D. Previous research has demonstrated positive effects of pharmaceutical support in training on correct pen handling [38,39,40,41]. Such interventions should emphasize hands-on, e.g., simulation-based, training to enable immediate feedback on incorrect technique.

### 4.5. Deficiencies Towards Seeking Medical Assistance

Given the potentially life-threatening nature of DKA, immediate hospital admission is recommended if ketosis home management is ineffective [2,3,4]. Nevertheless, more than two thirds of adolescents and almost half of caregivers reported not seeking any form of medical assistance.

In accordance with guidelines, adolescents and caregivers living with T1D received written guidance (ketosis management plan) and access to an emergency hotline to support ketosis home management, both provided by the pediatric diabetes department [2,4,7,43]. Despite this, only 13% of adolescents and fewer than half of the caregivers in our study reported contacting the emergency hotline. Similarly, fewer than 25% of adolescents used the ketosis management plan during this study’s standardized clinical case scenario. However, those participants using the plan reported more management steps in the standardized clinical case scenario. These findings underscore the importance of readily accessible support for ketosis home management, which should be more actively promoted by health care professionals [7,16,43].

### 4.6. Mismatch Between Subjective Self-Ratings and Objective Assessment

Both adolescents and caregivers provided high subjective self-ratings on their competence in ketosis home management, which exceeded self-reports described in prior studies [32]. Together with the generally low reported desire for ketosis/DKA prevention training, these findings suggest that adolescents and caregivers perceive themselves as competent in preventing DKA and see little need for additional support or improvement.

Contrasting these findings with the objectively assessed challenges in ketosis home management reveals a mismatch between participants’ self-perception and objective assessment. Such mismatches have similarly been shown in the management of severe hypoglycemia and in the administration of anticonvulsive rescue medication [44,45].

The observed mismatch may partly reflect a Dunning–Kruger effect which describes a cognitive bias in which individuals with low competence in a certain domain tend to overestimate their own abilities in this domain [46]. Partly, the mismatch may also reflect a limited awareness of correct handling. Unawareness of correct handling may hinder the recognition of handling errors which may lead to limited interest in training and improvement.

To improve competence in ketosis home management and thus potentially in preventing DKA, adolescents and caregivers require guidance and training by health care professionals. Regular hands-on ketosis/DKA prevention training, focusing on behavioral interventions and involving pharmacists and diabetes specialists, has proven to be efficient [14,15,16]. To address the challenges identified in this study, training programs should include clinical case scenarios and simulation-based practical demonstrations for repeated competency assessment. Furthermore, error-based learning strategies with deliberately embedded errors to be recognized by participants could be employed. Combining these concepts with teach-back techniques in which participants explain or demonstrate their understanding in their own words to verify comprehension, may improve ketosis home management skills. To address limited motivation to engage in ketosis/DKA prevention training among individuals living with T1D, such training should be proactively offered by health care professionals. Moreover, published evidence indicates that ketosis/DKA awareness campaigns enhance recognition of ketosis and DKA and thereby support its prevention [47,48,49,50].

## 5. Conclusions

This exploratory study revealed challenges in ketosis home management aimed at preventing DKA. In particular, ketone self-testing, insulin administration and seeking medical assistance were found to be challenging. The mismatch between objective assessment and high subjective self-ratings on competence in ketosis home management is a potential barrier to ideal glycemic control. To address this barrier, recurrent patient-centered training and comprehensive ketosis/DKA awareness campaigns are essential.

## 6. Limitations

Limitations of this study include its single-center design and locally specific training protocol, which limits generalizability beyond the specific setting. Voluntary participation may have led to overrepresentation of confident individuals, and subjective self-assessments are prone to bias. Practical skills were assessed using demonstration models rather than monitoring during actual impending DKA episodes. Evaluation of the household availability of urine dipsticks and insulin cartridges via telephone, when home visits were declined, may have led to inaccuracies. Data collection was performed by a trained pharmacist as a single evaluator. While this approach ensures consistency and minimizes interviewer bias, it poses a risk for observer and confirmation bias which limits the study’s generalizability.

## Figures and Tables

**Table 1:** Domains, questions and management steps of the standardized clinical case scenario to objectively assess competence in ketosis home management. DKA: Diabetic ketoacidosis.

Domain	Question	Management Step
Domain A: Initiation of ketosis home management	Your/your child’s blood glucose level is at 22.0 mmol/L and nausea or abdominal pain is present. What would you do?	(1) Perform ketone self-testing (in blood or urine)
Domain B: Implementation of ketosis home management	The ketone test showed elevated ketone levels (urine: ++++, blood: 1.7 mmol/L). What would you do?	(2) Calculate the increased insulin dose (200% of the usual correction bolus) Optional: seek medical assistance
		(3) Administer insulin Optional: seek medical assistance
		(4) Use a pen or a syringe for insulin administration (do not use an insulin pump) Optional: seek medical assistance
	How would you proceed after insulin administration?	(5) Check blood glucose levels regularly (at least every 2 h) Optional: seek medical assistance
		(6) Check ketone levels regularly (at least every 2 h) Optional: seek medical assistance
		(7) Stay hydrated (with oral, sugar-free fluids) Optional: seek medical assistance
		(8) Check for pen or pump malfunctions Optional: seek medical assistance
Domain C: Termination of ketosis home management	2 h after administering 200% correction bolus, the blood glucose level is at 16.0 mmol/L, and the ketone levels are elevated (urine: ++++, blood: 1.7 mmol/L). What would you do?	(9) Mandatory: Seek immediate medical assistance if ketone and/or blood glucose levels remain elevated
	When would you consider DKA averted?	(10) Consider DKA averted if there are no ketones present in blood or urine

++++ Symbol indicating very high ketone levels.

**Table 2:** Demographic data of enrolled adolescents and caregivers.

Characteristic	Adolescents (N = 61)	Caregivers (N = 79)
Age [years]: median (Q25/Q75; min/max)	15 (13/16; 12/17)	39 (36/42; 23/69)
Gender [*n* (%)]		
Male	25 (41%)	16 (20%)
Female	36 (59%)	63 (80%)
Education [*n* (%)]		
Not yet in school	0 (0%)	-
Primary school	0 (0%)	-
Middle school	27 (44%)	-
Grammar school	31 (51%)	-
Special-needs school	1 (2%)	-
(Pre-)Vocational training	2 (3%)	-
Professional education [*n* (%)]		
University degree/university of applied sciences degree		27 (34%)
Completed vocational training		50 (63%)
Other		2 (3%)
Insulin therapy [*n* (%)]		
Insulin pen	36 (59%)	33 (42%)
Insulin pump	25 (41%)	46 (58%)
Time since diagnosis of diabetes [years]: median (Q25/Q75; min/max)	5 (3/8; 0.3/15)	3 (1/5; 0.1/11)
Time since last ketosis/DKA prevention training [years]: median (Q25/Q75; min/max)	1 (0.5/2; 0.1/4) ^a^	2 (0.6/3; 0.1/9) ^b^

^a^ A proportion of 41% (25/61) of participants gave no answer or answered “I don’t know”. ^b^ A proportion of 6% (5/79) of participants gave no answer or answered “I don’t know”.

**Table 3:** Subjective self-ratings of adolescents and caregivers on knowledge of the concept of DKA, ability to identify symptoms of DKA, and skills in managing DKA. Scale: non-existent (0), very poor (1), inadequate (2), acceptable (3), fair (4), good (5), very good (6). DKA: Diabetic ketoacidosis.

Item	Adolescents (N = 61)	Caregivers (N = 79)
Knowledge of the concept of DKA: [median (Q25/Q75; min/max)]	5 (4/6; 0/6)	6 (5/6; 1/6)
Ability to identify symptoms of DKA: [median (Q25/Q75; min/max)]	5 (3/6; 0/6)	5 (4/6; 2/6)
Skills in managing DKA: [median (Q25/Q75; min/max)]	5 (5/6; 0/6)	6 (5/6; 3/6)

**Table 4:** Handling errors objectively assessed in the practical demonstrations of urine dipstick self-testing.

Item	Adolescents (N = 47)	Caregivers (N = 62)
Number of handling errors per demonstration: [median (Q25/Q75; min/max)]	3 (3/4; 1/5)	3 (2/3; 0/5)
Excess test solution was not removed [*n* (%)]	40 (85%)	49 (79%)
Dipstick was not removed from test solution promptly [*n* (%)]	36 (77%)	42 (68%)
Test result was interpreted after inappropriate time [*n* (%)]	31 (66%)	39 (63%)
Dipstick container was not resealed immediately [*n* (%)]	27 (57%)	27 (44%)
Test area was touched [*n* (%)]	10 (21%)	11 (18%)
Test area was not fully immersed into test solution [*n* (%)]	1 (2%)	1 (2%)

**Table 5:** Documented handling errors during practical demonstrations of insulin administration via insulin pen.

Item	Adolescents (N = 41)	Caregivers (N = 44)
Number of handling errors per demonstration: [median (Q25/Q75; min/max)]	2 (1/4; 0/6)	2 (1/3; 0/5)
No or incorrect insulin pen safety test [*n* (%)]	39 (95%)	43 (98%)
Needle remained injected for less than 10 s [*n* (%)]	22 (54%)	15 (34%)
Needle was not removed from insulin pen after injection [*n* (%)]	16 (39%)	12 (27%)
Needle cap was not removed before injection [*n* (%)]	14 (34%)	14 (32%)
Needle was not attached to insulin pen before injection [*n* (%)]	11 (27%)	6 (14%)
No insulin was administered [*n* (%)]	2 (5%)	1 (2%)
Insulin dose was not set on insulin pen [*n* (%)]	1 (2%)	0 (0%)

## Data Availability

The datasets generated during and/or analyzed during the current study are not publicly available due to the privacy protection requirements of the study participants but are available from the corresponding author on reasonable request.

## Abbreviations

The following abbreviations are used in this manuscript:

DKA	Diabetic ketoacidosis
T1D	Type 1 diabetes
